# Root exudates drive the soil-borne legacy of aboveground pathogen infection

**DOI:** 10.1186/s40168-018-0537-x

**Published:** 2018-09-12

**Authors:** Jun Yuan, Jun Zhao, Tao Wen, Mengli Zhao, Rong Li, Pim Goossens, Qiwei Huang, Yang Bai, Jorge M. Vivanco, George A. Kowalchuk, Roeland L. Berendsen, Qirong Shen

**Affiliations:** 1 Jiangsu Provincial Key Lab for Organic Solid Waste Utilization; National Engineering Research Center for Organic-based Fertilizers; Jiangsu Collaborative Innovation Center for Solid Organic Waste Resource Utilization, Nanjing Agricultural University, Nanjing, 210095 China; 2 School of Geography Science, Nanjing Normal University, Nanjing, 210021 China; 3 Plant-Microbe Interactions, Institute of Environmental Biology, Utrecht University, Padualaan 8, 3584 CH Utrecht, the Netherlands; 4 State Key Laboratory of Plant Genomics, Institute of Genetics and Developmental Biology, Chinese Academy of Science, Beijing, 100101 China; 5 Department of Horticulture and Landscape Architecture and Center for Rhizosphere Biology, Colorado State University, Fort Collins, CO 80523 USA; 6 Ecology and Biodiversity Group, Department of Biology, Institute of Environmental Biology, Utrecht University, Padualaan 8, 3584 CH Utrecht, the Netherlands

**Keywords:** Soil-borne legacy, Foliar pathogen, Microbiome, Disease-suppressive soil, Root exudates

## Abstract

**Background:**

Plants are capable of building up beneficial rhizosphere communities as is evidenced by disease-suppressive soils. However, it is not known how and why soil bacterial communities are impacted by plant exposure to foliar pathogens and if such responses might improve plant performance in the presence of the pathogen. Here, we conditioned soil by growing multiple generations (five) of *Arabidopsis thaliana* inoculated aboveground with *Pseudomonas syringae* pv *tomato* (*Pst*) in the same soil. We then examined rhizosphere communities and plant performance in a subsequent generation (sixth) grown in pathogen-conditioned versus control-conditioned soil. Moreover, we assessed the role of altered root exudation profiles in shaping the root microbiome of infected plants.

**Results:**

Plants grown in conditioned soil showed increased levels of jasmonic acid and improved disease resistance. Illumina Miseq 16S rRNA gene tag sequencing revealed that both rhizosphere and bulk soil bacterial communities were altered by *Pst* infection. Infected plants exhibited significantly higher exudation of amino acids, nucleotides, and long-chain organic acids (LCOAs) (C > 6) and lower exudation levels for sugars, alcohols, and short-chain organic acids (SCOAs) (C ≤ 6). Interestingly, addition of exogenous amino acids and LCOA also elicited a disease-suppressive response.

**Conclusion:**

Collectively, our data suggest that plants can recruit beneficial rhizosphere communities via modification of plant exudation patterns in response to exposure to aboveground pathogens to the benefit of subsequent plant generations.

**Electronic supplementary material:**

The online version of this article (10.1186/s40168-018-0537-x) contains supplementary material, which is available to authorized users.

## Background

Plants are continually under attack from a variety of microbial pathogens that cause disease. However, some soils have the capacity to suppress plant disease even in the presence of a virulent pathogen and under climatic conditions that are favorable for disease development [1, 2]. Such naturally disease-suppressive soils have been reported for diseases across a diverse range of agricultural crops, such as Take-all and Rhizoctonia bare patch disease on wheat [1], potato common scab [3], *Fusarium* wilt on strawberry and vanilla [4, 5], and *Rhizoctonia solani* on sugar beet [6]. In some disease-suppressive soils, this suppressiveness is related to the abundance of specific microbes in the soil [4, 6]. In high abundance, beneficial microbes can directly inhibit pathogens by producing antimicrobial compounds. However, beneficial microbes can also inhibit pathogens indirectly by stimulating the plant’s immune system, a phenomenon called induced systemic resistance (ISR) [7]. In order for disease-suppressive communities to develop, successive cropping cycles of the same plant species need be grown in the presence of a severe disease outbreak. This observation has led to the hypothesis that, upon attack, plants enrich and sustain specific beneficial microbes that come to their aid [1, 8, 9]. Some evidence for this “cry for help” hypothesis is beginning to accumulate. In the case of wheat and pepper, defense activation has been demonstrated to stem from plant-mediated changes in rhizosphere microbial communities [10, 11]. In the model plant *Arabidopsis thaliana* (hereafter referred to as *Arabidopsis*), the infection of leaves by *Pseudomonas syringae* pathovar *tomato* DC3000 (hereafter referred to as *Pst*) has been shown to induce the secretion of malic acid by roots, which led to the promotion of the ISR-inducing *Bacillus subtilis* strain FB17 on gnotobiotic roots of infected plants. Moreover, it was shown recently that also in natural soils, *Arabidopsis* plants can promote a select group of beneficial microbes in the rhizosphere [12]. Upon foliar defense activation by the downy mildew *Hyaloperonospora arabidopsidis*, a beneficial consortium including a *Xanthomonas* sp., a *Stenotrophomonas* sp., and *Microbacterium* sp. populations was strongly promoted in the rhizosphere. Furthermore, upon isolation, these strains could together induce resistance to down mildew when inoculated back to *Arabidopsis*. Moreover, downy mildew infection in a first population of plants increased the resistance of a second population of plants growing in the same soil. Together, these results indicate that plants can recruit beneficial microbes upon attack to generate a soil memory or “soil-borne legacy” that better prepares the next generation of plants to avoid harmful effects of the pathogen [12–15]. In this process, root exudates and other root-derived molecules are believed to play a role [12, 13, 16–18], although direct evidence supporting this hypothesis is generally lacking. In this study, we examined the role of root exudates in the establishment of soil-borne legacies following foliar pathogen attack. We conditioned soils by growing five successive generations of *Arabidopsis* in the same soil. In each generation, plants were either infected or not infected with *Pst*. In this way, we generated pathogen-conditioned soils as well as control-conditioned soils. We subsequently examined plant growth and hormone production in the following (sixth) generation in the absence of the pathogen. We also tracked the soil bacterial community in the bulk as well as the rhizosphere soil by high-throughput 16S rRNA gene tag sequencing. Concurrently, we identified compounds that were differentially exuded by infected versus uninfected plants and tested these compounds for their ability to promote disease suppressiveness in soil. Finally, we examined whether these compounds affect plant resistance directly or indirectly through their effects on the microbiome. By combining these complementary lines of investigation (Additional file 1: Figure S1), we were able to examine how changing exudation patterns can act as a mechanism by which plants can build their soil-borne legacy to the benefit of future plant generations.

## Methods

### Development of pathogen-conditioned soils

The soil used in this experiment was collected in July 2014 from a site near the Michigan Extension Station (Benton Harbor: N 42°05′34″, W 86°21′19″) where *Arabidopsis thaliana* genotype Pna-10 has grown naturally for more than a decade. Soils from this site have been used in several previous studies [16, 19, 20]. The collected soil was transported to the laboratory in airtight coolers and stored in a cold room (4 °C) until use. Before the start of the experiment, all soils were dried at room temperature, pooled, and thoroughly sieved to remove roots and other plant tissue.

To condition soils for five generations, *Arabidopsis thaliana* accession Col-0 plants were first sown on Murashige and Skoog [21] ager plates supplemented with 1% sucrose stratified for 2 days at 4 °C and allowed to germinate and grow in a climate chamber (25 ± 2 °C, 16 h light/8 h dark, light intensity 45 μmol m s^−1^; these conditions were used for plant growth throughout this study). Four 7-day-old seedlings were transferred to each of 108 pots (3 pots per set, 18 replicate sets for each treatment). Each pot contained 50 g of soil. The plants in half of the pots (54 pots) were inoculated with *Pst* at 14 days post-transplantation. Four true leaves of each plant (16 leaves per pot) were punctured using a syringe needle and 1 μL of a *Pst* suspension (10^7^ CFU/mL, *Pst* was pre-cultured overnight in nutrient broth at 37 °C with 170 rpm shaking and cell density adjusted by addition of sterilized water) was added to the wound. Special care was taken to avoid contamination of the soil with the *Pst* inoculum. Sterilized water was added to the punctures in control-treated plants (the other 54 pots). After the *Pst* suspensions had air-dried on the leaves, all pots were randomized, placed back into the growth chamber, and covered to maintain high humidity. *Pst* infections were allowed to develop for 14 days, at which time wounded leaves became diseased as evidenced by a clearly visible necrotic area. Subsequently, the aboveground plant parts, but not the roots, were removed, and the pots containing the soil were air-dried for 1 week. Subsequently, 5 mL of MS medium was added to each pot and new 7-day-old seedlings were transplanted to the pots for the next generation. This process was repeated for a total of five consecutive plant generations with the same batch of seeds (Additional file 1: Figure S1).

### Assessment of disease resistance on conditioned soils

One 7-day-old *Arabidopsis* seedling was transferred to the center of each of the 27 pathogen-conditioned and 27 control-conditioned pots (conditioned as described above) and incubated in a climate chamber. Fourteen days after transplantation, all plants were inoculated with *Pst* as described above. Seven days after inoculation, the number of necrotic leaves as a percentage of the total number of inoculated leaves was recorded to determine the disease incidence.

### Microbiome sample collection and determination of phytohormone levels in the absence of the pathogen

The remaining conditioned soils (27 pots for each of the two treatments) were used to assess the effect of conditioning on phytohormone levels and microbiome composition in the absence of the pathogen. Again, one 7-day-old *Arabidopsis* seedling was transferred to the center of each conditioned pot. After 28 days of growth, aboveground plant biomass was harvested, weighed, and analyzed for phytohormone content. Rhizosphere soils were collected as described previously [19]. Briefly, roots were gently removed from soil together with the tightly adhering soil. The bulk soil was also collected after the rhizosphere soil collection (remaining soil). Within each treatment, samples from three randomly selected pots were pooled, thereby yielding nine composite samples for rhizosphere and bulk soils for both the pathogen-conditioned and control-conditioned soils. All samples were immediately stored at − 80 °C prior to subsequent DNA analysis.

Phytohormones in shoot tissue were extracted from 100 mg pre-ground aboveground fresh tissue in 1 mL cold extraction solvent (20:79:1, methanol: isopropanol: acetic acid, v:v:v). Ten microliters of internal standard solution (jasmonic acid (JA): 10 ng/mL, salicylic acid (SA) 60 ng/mL, and abscisic acid (ABA) 10 ng/mL) was added to each sample. The extraction process and LC-MS/MS analysis were performed as previously described [22]. Specifically, tandem mass spectrometry coupled to liquid chromatography was performed on a Waters Xevo TQ-S triple quadruple mass spectrometer coupled to a Waters M-class Acquity UPLC system. Optimization of parent ion, cone voltage, collision energies, and fragment ions was done via direct infusion of phytohormones into the mass spectrometer for selected reaction transitions. LC-MS/MS buffers were as follows: buffer A was water with 0.1% formic acid and buffer B consisted of acetonitrile with 0.1% formic acid. Buffers were added in a gradient as follows: 10% buffer B was used to start, at 8 min 97% buffer B, and at 9.5 min 10% buffer B. We used a Waters 3 μM Atlantis dC18 (300 μM× 150 mm) column set to a flow rate of 11.5 μL/min with column temperature held at 40 °C. Injection volume was 1 μL and autosampler temperature was set at 4 °C. Waters TargetLynx software was used for data analysis and hormone quantification. Quantification of phytohormones was done with the following formula: analyte peak area × (internal standard concentration/internal standard peak area). By utilizing polarity switching, all samples were analyzed via LC-MS in a single run with dwell times of 10 ms. In positive ion mode, capillary voltage was 3.2 kV, while in negative ion mode, it was − 2.2 kV. Nebulizer gas flow was 7.0 bar, cone gas flow was 150 L/h, desolvation temperature was 225 °C, and desolvation gas flow was 825 L/h. Argon was used as collision gas at a flow rate of 0.2 mL/min.

### PCR amplification and sequencing

Total DNA was extracted from a total of 18 rhizosphere samples (9 for each treatment) and 18 bulk soil samples. Extractions were carried out on 500 mg of soil (wet weight) using the Power Soil DNA Isolation kit (Mo Bio Laboratories Inc., Carlsbad, CA, USA), according to the manufacturer’s instructions. DNA was extracted from two technical replicates per sample to minimize the DNA extraction bias. Samples were stored at − 20 °C, and technical replicates were pooled before performing polymerase chain reaction. The DNA quality was assessed according to the 260/280-nm and 260/230-nm absorbance ratios using a NanoDrop ND-2000 spectrophotometer (NanoDrop, ND2000, Thermo Scientific, 111 Wilmington, DE). The concentration of extracted DNA was between 40 and 60 ng/μL. Bacterial 16S rRNA gene fragments were amplified from the extracted DNA using primers 341F (CCTAYGGGRBGCASCAG) and 806R (GGACTACHVGGGTWTCTAAT) and the following PCR conditions: 30 s at 95 °C, 30 s at 59 °C, and 30 s at 72 °C for 30 cycles. PCRs were performed in a total volume of 25 μL with 9.75 μL water, 5 μL 5× PCR buffer, 5 μL 5× Q5 GC high enhancer, 2 μL deoxynucleoside triphosphates (dNTPs), 1 μL of each primer, 0.25 μL and 5 U/μL of Q5 polymerase, and 1 μL of extracted DNA [23]. After PCR amplification, bands were excised and purified from 1.5% agarose gels using the MinElute PCR Purification Kit (Qiagen, Germany) and the QiagenQIAquick Gel Extraction kit (Qiagen, Germany). Amplicons were subjected to paired-end sequencing on the Illumina MiSeq sequencing platform using PE250 chemical at the Genomics Core of Michigan State University. After assembly, we got ~ 480 bp reads covering the V3–V4 region of the bacterial 16S rRNA.

### Amplicon sequence processing and analysis

Amplicon sequences were analyzed using the Qiime2 environment (version 2017.12, https://qiime2.org/). Initial sequence quality was assessed using the “Demux” plugin. Due to low sequence quality at the 3′-ends of the reads, joining paired-ends resulted in unreliable reads and greatly reduced the amount of paired-end reads that passed quality control. We therefore only used the forward reads (containing the relatively short but highly discriminating V3 region) and employed the DADA2 pipeline [24]. Sequences were truncated at base 140 and trimmed until base 17. This resulted in relatively short reads (~ 120 bp) of high quality from which actual sequence variants (ASVs) were identified. The DADA2 pipeline then produced an ASV count table containing 1.35 million usable reads and ~ 5000 ASVs. In some cases, we observed ASVs that were highly abundant in one sample but absent from the rest of the dataset. These were judged to likely be from chimeric sequences that were not filtered out in the DADA2 pipeline (as a consequence of using only the forward reads) and excluded from further analysis. The resulting final ASV table contained ~ 900,000 high-quality reads belonging to ~ 2000 ASVs. Taxonomic assignment of ASVs was performed using the VSEARCH consensus taxonomy classifier implemented in Qiime2 and the SILVA 16S rRNA database [25]. Statistical analyses of the 16S rRNA microbiome sequencing data was performed using the Qiime2 environment (version 2017.12) and in the R environment (R 2017, https://www.r-project.org version 3.4.3). β-diversity (PCoA based on Bray-Curtis dissimilarities) was calculated using the “phyloseq” package (version 1.22.3) [26]. Statistical significance of the β-diversity between treatments was calculated through analysis of similarity (ANOSIM), as implemented in the Qiime2 environment. Graphs of the microbiome data were created using the “ggplot2” package (version 2.2.1). The code used in this process was listed in Additional file 1.

### *Pst* detection in soil by PCR

The detection of *Pst* was carried out using the PCR primers OWB575 (AACTGAAAAACACCTTGGGC) and OWB576 (CCTGGGTTGTTGAAGTGGTA) that target the *Oprf* gene of *P*. *syringae* [27]. PCR conditions were as follows: an initial 95 °C for 4 min and 30 cycles of 30 s at 95 °C, 30 s at 59 °C, and 30 s at 72 °C. PCRs were performed in a total volume of 25 μL with 17.2 μL water, 2.5 μL 10× PCR buffer (Mg^2+^ plus), 2 μL deoxynucleoside triphosphates (dNTPs), 1 μL of each primer, 0.3 μL Taq polymerase (TaKaRa Biotechnology Co., Ltd), and 1 μL of extracted DNA. Water was used as the negative control, and genomic DNA extract from *Pst* was the positive control. The PCR products were visualized by electrophoresis through a 1% agarose gel.

### Soil chemical analyses

The soil chemical analyses were conducted on 5 g of bulk soil collected at the sixth generation of plant growth after the aboveground plant parts were removed. Both conditioned and control soil samples were used to analyze available phosphorus (AP), available potassium (AK), nitrate (NO_3_^−^), ammonia (NH_4_^+^), and pH [28]. All analyses were performed at the Soil, Water and Plant Testing Laboratory at Colorado State University.

### Root exudate collection and GC-MS analysis

For root exudate collection, surface-sterilized *Arabidopsis* (Col-0) seeds were placed on MS agar-solidified medium (1% agar) amended with 3% sucrose, stratified for 2 days at 4 °C, and allowed to germinate and grow for 14 days in a climate chamber. Plants were subsequently inoculated with *Pst* as described above and, after the pathogen suspension had air-dried on the leaves, plants were transferred to 6-well plates containing water-agar medium (1% agar) for exudate collection. Plates were randomized during the collection period. Each treatment contained three replicate plates with six plants. Non-inoculated but wounded plants were used as control.

Plates were covered and incubated in the climate chamber for 3 days. After 3 days of collection, the water-agar medium of the 6 wells in one plate were pooled as one sample and lyophilized for further extraction. Root exudates were extracted from the lyophilized agar medium with 80% methanol for further extraction and GC-MS analyses were performed as previously described [16]. Briefly, extracts were dried under nitrogen gas and then methoximated and trimethylsilylated. The derivatives were analyzed by an Agilent 6890 gas chromatograph (Santa Clara, CA) containing a 30-m-long, 0.25-mm inner diameter rtx5Sil-MS column with an additional 10-m integrated guard column. Metabolites were detected using the BinBase algorithm [29] and identified by comparing the retention index and mass spectrum of each analyte against the Fiehn mass spectral library from the West Coast Metabolomics Center, University of California Davis. The experiment was repeated and compounds were analyzed using the same method at BIOTREE technology Co. Ltd. in Shanghai, China, and similar results were obtained.

### Impacts of root exudate classes on soil microbiome feedbacks to plant defense

To examine the effect of root exudates secreted upon aboveground *Pst* infection on soil suppressiveness, we selected four categories of differentially secreted exudates: long-chain organic acids (LCOAs), amino acids (AAs), short-chain organic acids (SCOAs), and sugars. For each exudate category, representative compounds were selected based on their altered abundance in root exudates after *Pst* infection. For the AA, SCOA, and sugar category, watery solutions were prepared containing each of the selected compounds in equal dosage and to a final total concentration of 10 mM. For LCOAs, the total concentration was 10 μM due to their lower solubility. The LCOA solution contained 2.0 μM pentadecanoic acid, 2.0 μM hexadecanoic acid, 2.0 μM palmitoleic acid, 2.0 μM octadecanoic acid, and 2.0 μM arachidic acid. The AA solution contained 1.67 mM isoleucine, 1.67 mM leucine, 1.67 mM methionine, 1.67 mM proline, 1.67 mM tryptophan, and 1.67 mM ornithine. The SCOA solution contained 2.5 mM citric acid, 2.5 mM aconitic acid, 2.5 mM succinic acid, and 2.5 mM malic acid. The sugar solution contained 1.67 mM maltose, 1.67 mM ribose, 1.67 mM glucose, 1.67 mM sucrose, 1.67 mM fructose, and 1.67 mM xylose.

Fifteen grams of soil was placed into each well of a 6-well plate. Plates were pre-incubated in a growth chamber at 30 °C for 1 week to allow the soil microbiome to acclimatize and to remove seedlings from the naturally occurring seedbank. Each well then received 1.5 mL of exudate compound solution twice a week for 8 ½ weeks (17 total applications) in a growth chamber at 30 °C. We applied three treatments: (1) L + A, equal volumes of the LCOA and AA solutions (representing pathogen-induced root exudates); (2) S + S, equal volumes of SCOA and sugar solutions (representing pathogen-repressed root exudates); and (3) water control. Each treatment consisted of 18 replicates divided over 3 plates. All plates were randomly placed during the incubation period.

To examine the effect of exudate compound classes on the development of soil suppressiveness, soil slurries were prepared and filter-sterilized as described previously [22]. Briefly, 5 g of each soil treatment was mixed with 50 mL autoclaved water on an orbital shaker for 1 h. After settling for an additional hour, soil slurry was obtained from the supernatant through filter paper and filtered slurry were filter-sterilized using a 0.22-μm filter polytetrafluoroethylene (PTFE) membrane. Slurries were prepared of the LA, SS, control-treated soils, and mixtures of LA and SS treated soils at ratios of 9:1, 5:5, and 1:9 (*v*/*v*). Seven-day-old *Arabidopsis* seedlings (prepared as described above) were transplanted to an autoclaved mixture of vermiculite and sand (volume: volume = 1:1) in 6-well plates to which 2 mL of soil slurry, filter-sterilized soil slurry, or autoclaved water was added. Five milliliters of MS medium was added to supply plants with the necessary nutrition during plant growth period. After 2 weeks, the *Pst* strain was inoculated onto the *Arabidopsis* leaves as described above. Disease incidence was determined 7 days after infection.

### Statistical methods

Statistically significant differences (*p* < 0.05) in disease incidence, plant biomass, phytohormones, root exudate compound abundance, and soil properties between controls and treatments were evaluated by Student’s *t* test or ANOVA using SPSS. The differences in bacterial community composition or in the root exudate composition among treatments were tested using PERMANOVA (Adonis, transformed data by Bray-Curtis, permutation = 999), implemented in R version 3.4.3.

The DESeq function of the “DESeq2” package (version 1.18.1) [30] was employed to test for differentially abundant ASVs in pathogen- and control-conditioned bulk soil and rhizosphere samples. Statistical significance was based on *p* value < 0.05 (with FDR < 5% under the Benjamini-Hochberg correction).

## Results and discussion

### Effect of pathogen-conditioned soil on plant performance

**Fig. 1:**
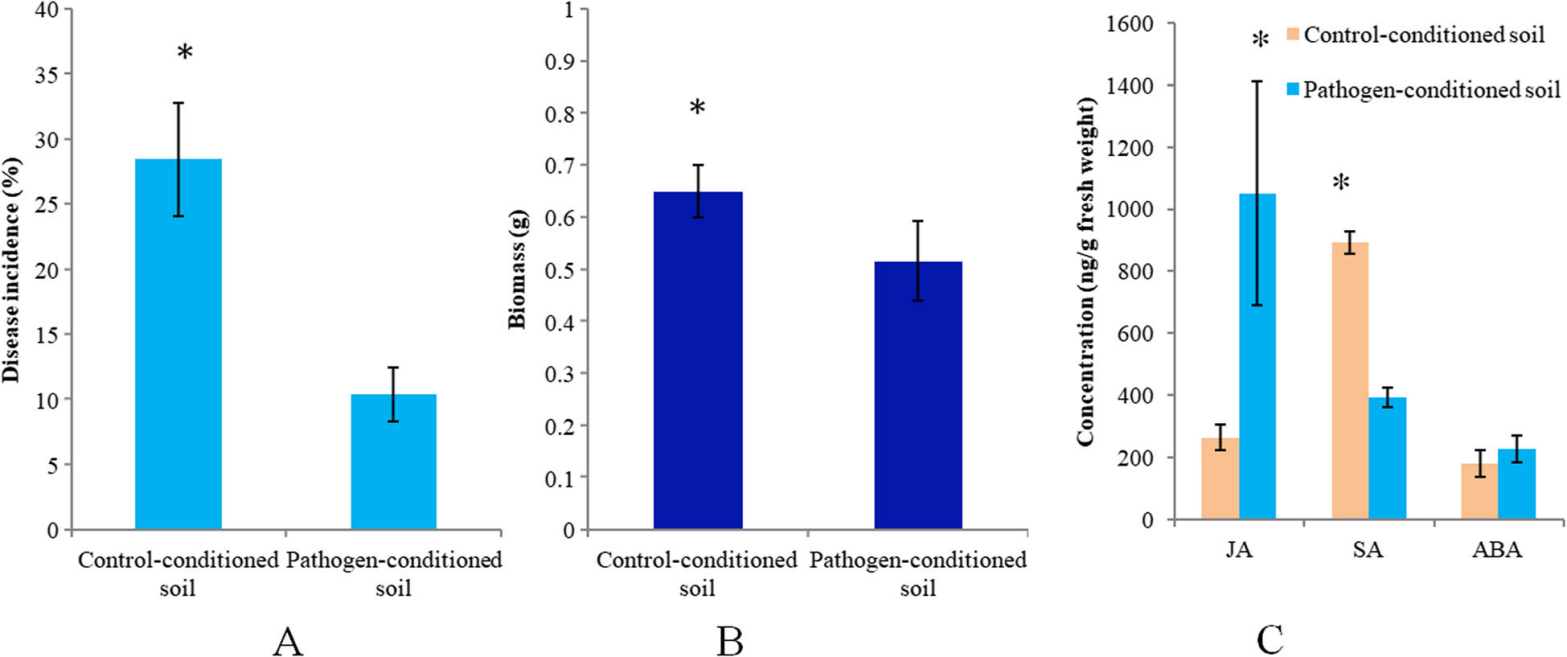
**a** Disease incidence after *Pst* inoculation of a sixth generation of *Arabidopsis* plants growing on control or pathogen-conditioned soils. The asterisk indicates statistically significant differences as determined with a Student’s *t* test (*p* < 0.05) between treatments. Bars represent the average of three replicates and error bars show standard deviations. **b** Fresh shoot weight of unchallenged *Arabidopsis* plants growing on control or pathogen-conditioned soil. **c** Concentrations of the phytohormones jasmonic acid (JA), salicylic acid (SA), and abscisic acid (ABA) in shoots of unchallenged *Arabidopsis* plants growing on control soil or pathogen-conditioned soil. The asterisk indicates statistically significant differences between treatments as determined with a Student’s *t* test, (*p* < 0.05). Bars represent the average of three replicates and error bars show standard deviations

Soils were conditioned by five succeeding generations of *Arabidopsis* plants of which the leaves were either infected by *Pst* (pathogen-conditioned soil) or subjected to a mock treatment (control soil). When a sixth generation of plants was planted on these conditioned soils and confronted with *Pst*, plants grown in pathogen-conditioned soils developed significantly (*p* < 0.05) reduced disease symptoms as compared to plants grown in control-conditioned soil (Fig. 1a). This supports previous findings that aboveground pathogen infection of a population of plants leads to a soil-borne legacy that induces resistance in a following population of plants growing in the same soil [12, 14].

In the absence of pathogen, plants grown in pathogen-conditioned soil exhibited significantly (*p* < 0.05) reduced fresh biomass compared to plants grown in the control soil (Fig. 1b). It is known that the activation of defenses comes at a cost for plant performance in the absence of the pathogen [31]. It is thus likely that the observed growth depression on pathogen-conditioned soil was the result of a redirection of plant metabolism toward defense at a cost of growth [32]. It should be noted that we did not detect any significant differences (*p* > 0.05, *t* test) in pH and nutrient content (NH_4_^+^, NO_3_^−^, AP, and AK) between the pathogen-conditioned and control soils (Additional file 1: Table S1). Thus, although we cannot rule out that there are other chemical differences between the soils of the two treatments, our data suggests that nutrient availabilities were not driving differences in plant growth in response to soil conditioning.

We then compared levels of the defense-regulating phytohormones jasmonic acid (JA), salicylic acid (SA), and abscisic acid (ABA) in unchallenged plants growing on differently pre-conditioned soils (Fig. 1c). JA levels were significantly higher (*p* < 0.05) in plants grown in pathogen-conditioned soil, while SA concentrations were significantly lower (*p* < 0.05). No significant differences were observed for abscisic acid (ABA). This indicates that the soil-borne legacy brought about by previous generations of pathogen-challenged plants induced production of JA in a sixth generation of unchallenged plants leading to defense activation and increased resistance. This suggests that the higher concentration of jasmonic acid induced by pathogen-conditioned soils resulted in reduced SA levels, as the SA- and JA-signaling pathways are known to antagonize each other [33]. Although ABA is known to fine-tune defense responses, it is mostly involved in response to abiotic stress [34–36]. It was therefore not surprising to find that biological soil conditioning had no effect on ABA levels.

It is important to note that the increased resistance in pathogen-conditioned soil was most likely not the result of the presence of *Pst* in the soil. The pathogens as only introduced onto the leaves and the aboveground plant parts were removed after 14 days, thereby minimizing the degree to which the pathogen might enter the soil. Moreover, *Pst* abundance was below detection limits in soil throughout the experiment as tested by PCR (Additional file 1: Figure S2) and qPCR [37].

### Impacts of aboveground pathogen infection on soil bacterial communities

**Fig. 2:**
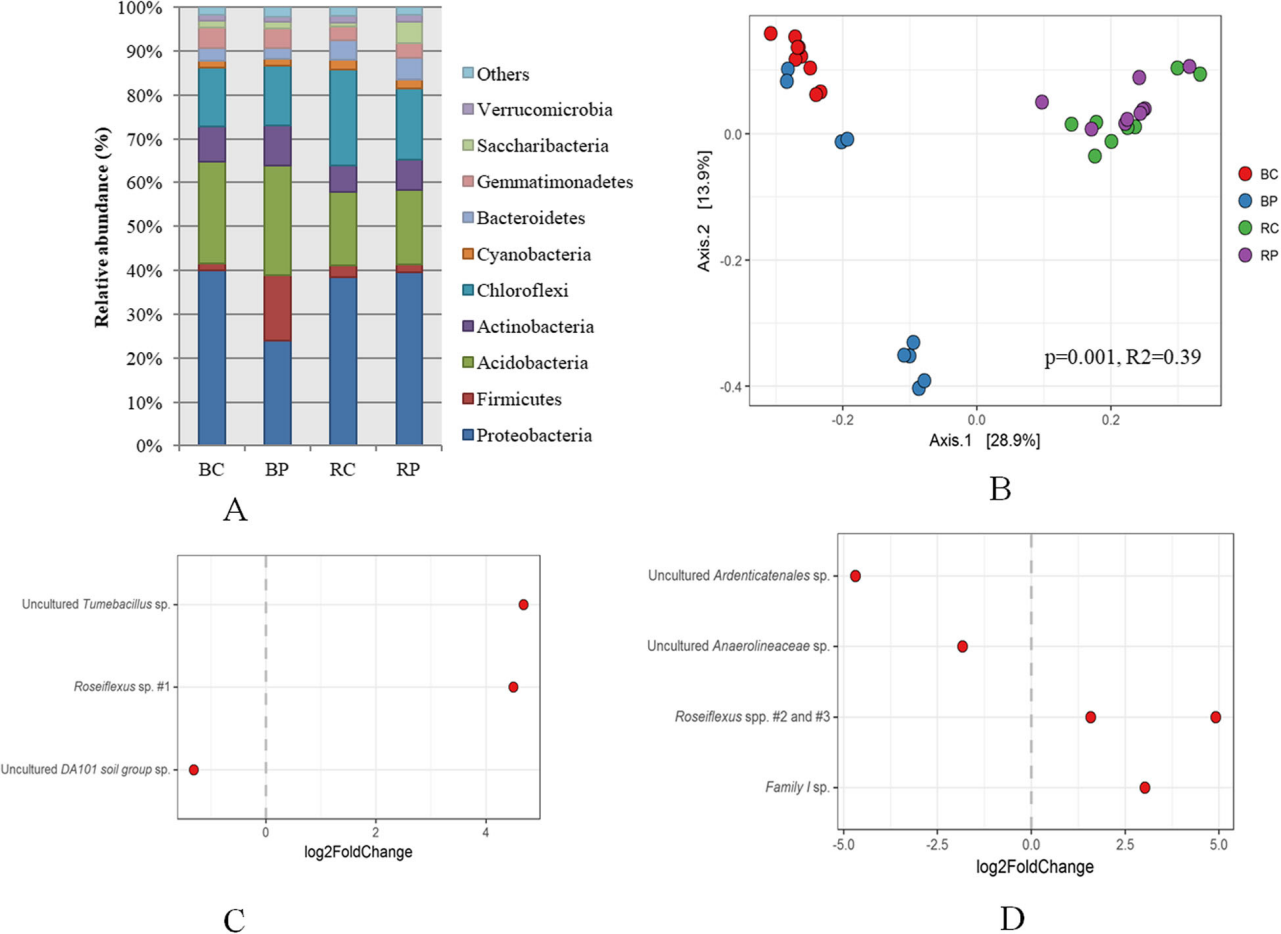
**a** Relative abundance (%) of the major bacterial phyla present in the microbial communities of control (C) or pathogen-conditioned (P) soils. Samples were taken of bulk (B) soil or rhizospheres (R) of unchallenged plants. **b** Principal coordinate analysis (PCoA) with Bray-Curtis dissimilarity of the microbial community in bulk soil (B) or rhizosphere (R) of control (C) or pathogen-conditioned (P) soils. **c** Relative abundance of the three ASVs that significantly differed between pathogen-conditioned and control-conditioned of the bulk soil. **d** Relative abundance of the five ASVs that significantly differed in relative abundance between pathogen-conditioned and control-conditioned of the rhizosphere soil

We analyzed the composition of microbial communities in the bulk soil and rhizosphere of unchallenged *Arabidopsis* plants growing on pathogen-conditioned or control soils using 16S rRNA gene amplicon sequencing. For this analysis, we collected 9 rhizospheres and 9 bulk soil samples from both pathogen-conditioned and control-conditioned soils, resulting to total of 36 microbial communities. We obtained an average read count per sample of 25,068 (standard deviation (SD) 6568). As typical of soils, bacterial communities were highly diverse as was reflected by the numbers of actual sequence variants (ASVs), generally ranged between 285 and 708 per sample with an average of 467 (SD 117). The majority of ASVs belonged to the phyla Proteobacteria (34.8%), Acidobacteria (20.6%), Chloroflexi (16.1%), Actinobacteria (7.6%), and Firmicutes (5.9%) (Fig. 2a). Principal coordinate analysis (PCoA) based on the detected ASVs showed a clear difference in community composition between bulk and rhizosphere soils, which was statistically significant as determined through analysis of similarity (PERMANOVA) (*p* = 0.001, *R*^2^ = 0.39) (Fig. 2b). This demonstrates a general rhizosphere effect of the plant [38, 39], even in soils that had been pre-conditioned by five generations of plant growth. Moreover, there was a statistically significant effect of the conditioning treatment in both the bulk soils (*p* = 0.001 in PERMANOVA, Additional file 1: Figure S3A) as well as the rhizosphere soils (*p* = 0.031 in PERMANOVA; Additional file 1: Figure S3B). Thus, aboveground *Pst* infections triggered a plant-mediated shift in belowground microbial community structure. The effect of aboveground infection, however, was more pronounced in bulk soil as compared to the rhizosphere, likely because we analyzed microbial communities only for unchallenged plants. We hypothesize that the rhizosphere effect of the unchallenged plants reduced our ability to observe a clear effect of pathogen conditioning. Upon closer inspection of the pathogen-conditioned bulk soils, these were relatively enriched in populations belonging to the Firmicutes, yet relatively depleted in Proteobacteria as compared to control soils (Fig. 2a). Furthermore, we detected that two ASVs, belonging to the genera *Fictibacillus* and *Sphingomonas*, respectively, seem to drive this separation, despite the fact that these ASVs did not differ significantly between the two treatments (*p* = 0.051 and 0.859 for the *Fictibacillus* ASV and the *Sphingomonas* ASV, respectively). The relative abundance of the *Fictibacillus* ASV was over 20% in five-out-of-nine pathogen-conditioned bulk soil samples, while it represented < 1% of the total community in the remaining four bulk soil samples and all of the rhizosphere samples (Additional file 1: Figure S4A and Table S2). Similarly, the relative abundance of the *Sphingomonas* ASV was greater than 11% in all bulk soil and rhizosphere samples except these same five pathogen-conditioned bulk soil samples in which the relative abundance was below 2% (Additional file 1: Figure S4B and Table S2). In a PCoA of these communities after the exclusion of these two ASVs, the separation of these five samples is no longer apparent, although the differences between the two conditioning treatments was still statistically significant (*p* = 0.001 in PERMANOVA, Additional file 1: Figure S5). The difference of average relative abundance of Firmicutes and Proteobacteria in bulk soils was also lost (Additional file 1: Figure S4C). Previously, it was found that aboveground downy mildew infection led to a very specific recruitment of three bacterial species to the roots of *Arabidopsis* [12]. Our data also show that only a very select number of ASVs is significantly impacted (*p* < 0.05) by the pathogen-conditioning soil treatment as compared to the control (Fig. 2c, d). Three ASVs differed between these treatments in the bulk soil (Fig. 2c) and five ASVs were significantly affected by the treatment in the rhizosphere samples (Fig. 2d). Of the ASVs affected by the treatment, five ASVs were more abundant in pathogen-conditioned soils, whereas three ASVs were less abundant. Remarkably, three ASVs that were significantly enriched in either pathogen-conditioned bulk or pathogen-conditioned rhizosphere soils belonged to the same genus, i.e., *Roseiflexus*.

These results thus appear to indicate that indeed only a few microbial populations are recruited by *Pst-*infected plants. However, even after removal of the most differential 200 ASVs, the remaining parts of the communities were still found to be significantly different according to PERMANOVA (Additional file 1: Table S3). This suggests that a large part of the microbial community contributes to the overall difference in community composition between the two soil compartments and two treatments. It is therefore difficult to pinpoint the extent to which specific ASVs, or combinations thereof, versus general community effects are responsible for the observed increase in plant resistance. However, it is clear that five generations of plants infected by *Pst* recruited a microbiome that was distinct from that of plants grown in the absence of the pathogen.

### Impact of *Pst* infection on root exudation profiles

**Fig. 3:**
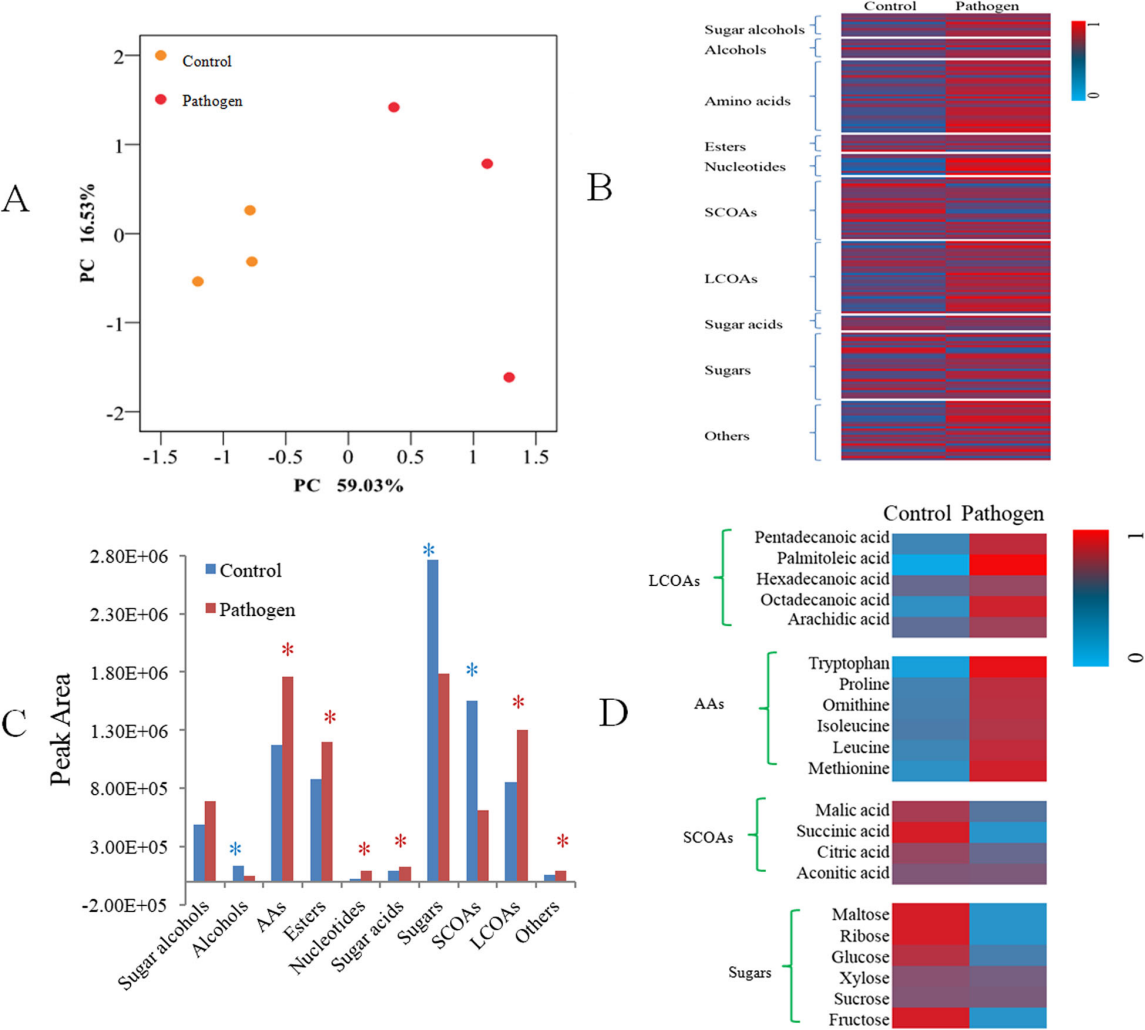
**a** Principal component analysis of root exudates of control-treated and *Pst-*inoculated plants growing on agar-solidified medium. **b** Heatmap analysis of changes in root exudate content of control-treated and *Pst-*inoculated plants growing on agar-solidified medium. **c** Abundance (cumulative peak area) of compound categories. Each bar represents the average of three replicates. The asterisk indicates statistically significant differences (*t* test, *p* < 0.05) between each root exudates of control-treated and *Pst-*inoculated plants. **d** Heatmap analysis of changes in abundance of representative compounds that were significantly differential in abundance between root exudates of control- and pathogen-treated plants and that selected for subsequent soil conditioning experiment. LCOAs long-chain carbon organic acids, SCOAs short-chain carbon organic acids, AAs amino acids

In order to establish a mechanistic explanation for how foliar infection of *Arabidopsis* alters the soil microbiome, root exudates of healthy and infected plants were collected in a gnotobiotic system and analyzed by gas chromatography–mass spectrometry (GC-MS). A total of 456 peaks were detected across all samples (Additional files 2). The overall exudation patterns from control plants were found to be distinct from those of plants infected with *Pst*, as demonstrated by their separation in a principal component analysis (PCA) (*p* = 0.043 in ANOSIM, Fig. 3a). The compounds belonging to 201 of the 456 detected peaks could be identified and placed into broad categories based on their chemical nature, namely sugars (31 compounds), sugar acids (7), sugar alcohols (11), short-chain organic acids (29), long-chain organic acids (34), nucleotides (10), amino acids (34), esters (8), alcohols (9), or others (28). All of the identified compounds were detected in both treatments, but the abundance of 50 compounds differed significantly (*t* test, *p* < 0.05) between the two treatments (Fig. 3b). When evaluated at the group level, alcohols, short-chain organic acids (SCOAs), and sugars were found to be significantly lower (*t* test, *p* < 0.05) in the control, while esters, amino acids (AAs), nucleotides, sugar acids, and long-chain organic acids (LCOAs) were significantly higher in abundance in root exudates from infected plants (Fig. 3c). Overall, *Pst* infection resulted in a significantly higher secretion of long-chain carbon compounds while secretion of small compounds was reduced (Fig. 3c). LCOAs and AAs were more abundant in root exudates after pathogen infection, whereas SCOAs and sugars were less abundant (Fig. 3c). It has been suggested that simple sugar exudates serve as non-selective C substrates [40], while more complex organic acids exert more selective effects [41]. Indeed, previous studies have been shown that SCOAs, especially those involved in the tricarboxylic acid cycle, can recruit PGPRs in the rhizosphere [13, 42]. It is thus noteworthy that long-chain organic acids were detected at higher concentrations in root exudates of infected plants.

### Impact of exudation compounds on disease suppression

**Fig. 4:**
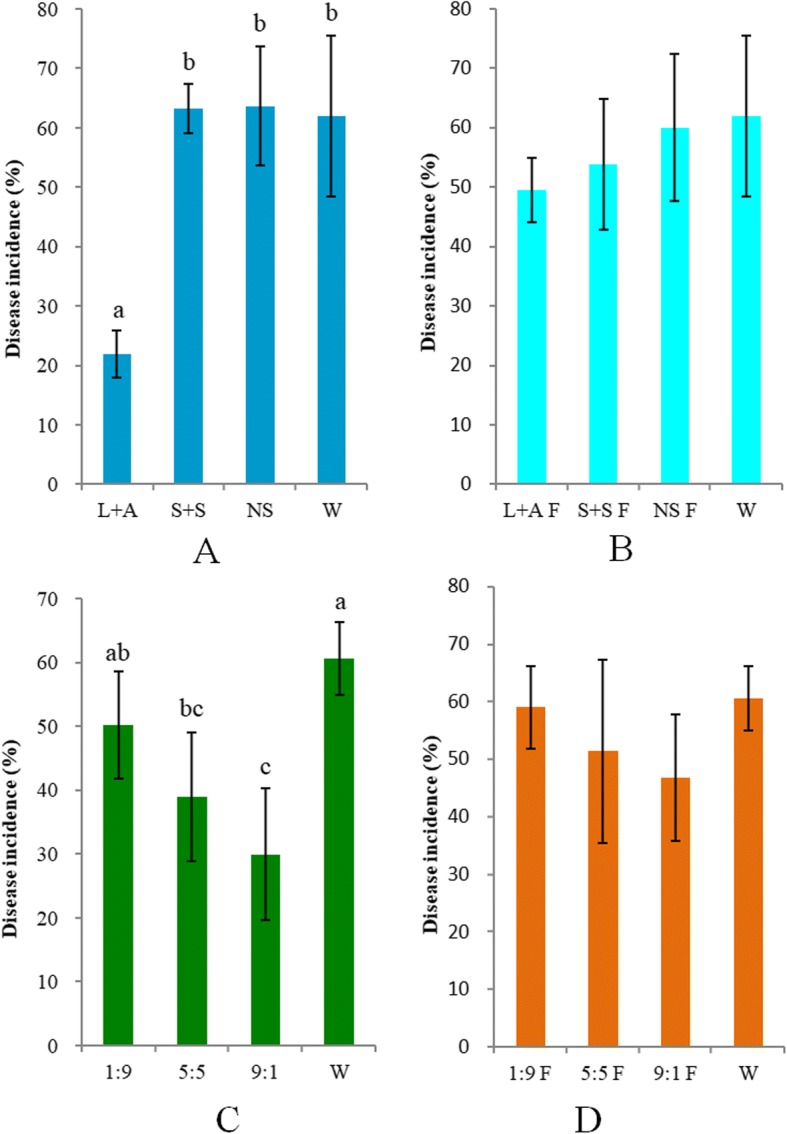
**a** Disease incidence of *Pst*-challenged *Arabidopsis* plants growing on sterilized vermiculite-sand mixtures inoculated with water (W), slurries of natural soil (NS) or slurries of soils preconditioned with mixtures of LCOAs and AAs (L + A), or SCOAs and sugars (S + S). **b** Disease incidence of *Pst*-challenged *Arabidopsis* plants growing on sterilized vermiculite-sand mixtures inoculated with filter-sterilized W, NS, L + A or S + S. **c** Disease incidence of *Pst*-challenged Arabidopsis plants growing on sterilized vermiculite-sand mixtures inoculated with W or L + A and S + S mixed in a ratio of 1:9, 5:5, or 9:1 (*v*/*v*). **d** Disease incidence of *Pst*-challenged Arabidopsis plants growing on sterilized vermiculite-sand mixtures inoculated with W or filter sterilized L + A and S + S mixed in a ratio of 1:9, 5:5, or 9:1 (*v*/*v*). Bars show average ± SD of six replicates. Different letters indicate significant (*p* < 0.05) difference according to ANOVA with Tukey’s post hoc test

To better understand the effect of differentially released compounds on soil microbes, we conditioned soil by repeatedly adding mixtures of compounds that were either over- or underrepresented in the exudation patterns of *Pst*-infected plants. To this end, exudate cocktails were made to represent four broad groups of differentially altered exudate compounds; LCOAs (pentadecanoic acid, hexadecanoic acid, palmitoleic acid, octadecanoic acid, and arachidic acid), the AAs (isoleucine, leucine, methionine, proline, tryptophan, and ornithine), SCOAs (citric acid, aconitic acid, succinic acid, and malic acid), and sugars (maltose, ribose, glucose, sucrose, fructose, and xylose) (Fig. 3d). Soil extracts were prepared from these pre-conditioned soils and used to inoculate naïve *Arabidopsis* plants grown in a sterilized mixture of sand and vermiculite, for subsequent challenge with *Pst*. The microbiome of soil preconditioned with mixtures of LCOAs and AAs (L + A) soils provided a greater level of induced resistance against foliar *Pst* than the microbiomes of control soils or soils pre-treated with SCOAs and sugars (S + S) (Fig. 4a). Importantly, induction of disease resistance was lost in those soil extracts in which bacteria had been removed by filtration (Fig. 4b), indicating that the microbiomes, rather than exudation compounds themselves, were eliciting the resistance in *Arabidopsis*.

Soil transfer experiments were also preformed to assess the amount of microbiome addition needed to confer resistance, using different amounts (10, 50, and 90% *w*/*w*) of L + A soil mixed into S + S soil before soil slurry preparation. At least partial disease suppressiveness was conferred by the addition of 50% and 90% microbiome from L + A soils to those of S + S soils (Fig. 4c). Predictably, none of the filtered soil, and thus microbe-free, slurries were able to confer disease suppressiveness (Fig. 4d). Collectively, these results indicated that disease suppressiveness toward *Pst* was microbiologically induced in soils conditioned by L + A mixtures.

## Conclusions

In this study, we found that infections by the foliar pathogen *Pst* triggered a soil-borne legacy that induced resistance in a following generation of plants. This soil-borne legacy was reflected by distinct bacterial communities in soils preconditioned by five generation of *Pst*-infected plants as compared to soils conditioned by non-inoculated plants. Moreover, when *Arabidopsis thaliana* was challenged by the foliar pathogen *Pst*, plant exudation patterns were altered and root secretions of LCOAs and AAs were increased. Application of a mixture of LCOAs and AAs to soil was sufficient to induce a similar soil microbiome-mediated pathogen-suppressive response as observed after for actual response to the pathogen.

**Fig. 5:**
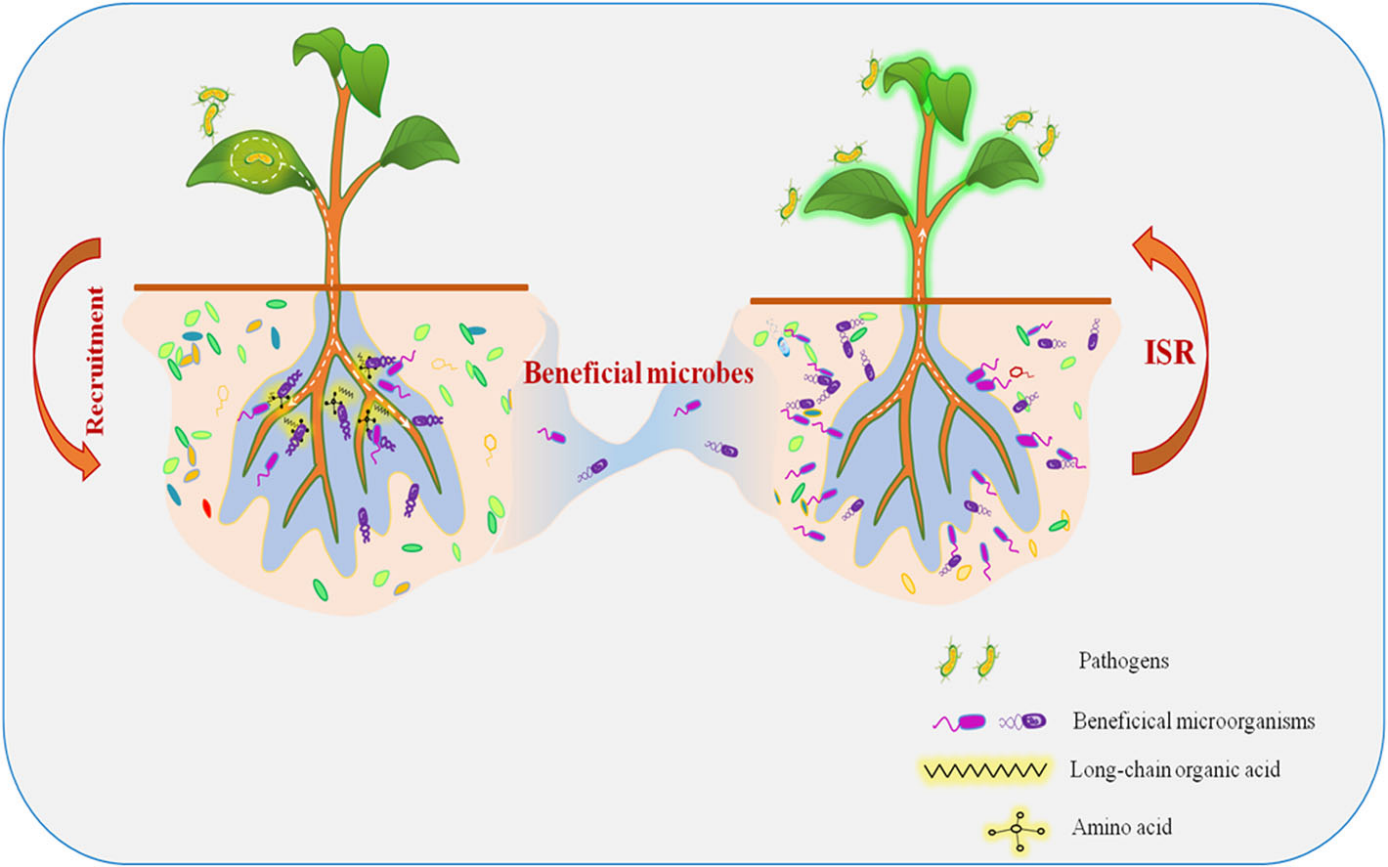
Mechanistic model of soil-borne legacies induced by foliar pathogens. First, the predecessor plants release root exudates into soil to manipulate soil microbial community dynamics and recruit beneficial microbes when attacked by foliar pathogens. The resulting shifts then elicit phenotypic changes (such as phytohormone level) in the new plants to adapt to the pathogens’ attack

Our combined results allow us to put forth a clear model related to responses to the aboveground pathogen (Fig. 5). We hypothesize that, upon infection, plant-systemic signaling leads to a change in root exudation profiles. These altered exudations in turn promote specific elements of the microbiome that induce resistance for future generations of the plant. Together, our results show not only that aboveground infection by the model bacterial pathogen *Pst* on the model plant *Arabidopsis thaliana* can have an effect on the resistance of subsequent plant populations growing in the same soil, but they also shed light on the mechanisms through which this soil-borne legacy is generated.

## Additional files

Additional file 1: **Figure S1.** Schematic representation of the experimental design. **Figure S2.** Detection of *Pseudomonas syringae* pv. tomato strain DC3000 by PCR. The length of target fragment is 304 bp. **Figure S3.** Principal coordinate analysis (PCoA) with Bray-Curtis dissimilarity of the microbial community in bulk soil (B) or rhizosphere (R) of control (C) or pathogen-conditioned (P) soils. A) bulk soils, B) rhizosphere soils. **Figure S4.** A and B are the relative abundance (%) of the bacterial genera within the *Firmicutes* and *Proteobacteria* phyla in the microbial communities of control and pathogen- conditioned bulk soils, respectively. C: Relative abundance (%) of the major bacterial phyla using the whole ASV table excluding ten differential ASVs (*Fictibacillus* and *Sphingomonas*) present in the microbial communities of control (C) or pathogen-conditioned (P) soils. Samples were taken from the bulk (B) soil or rhizospheres (R) of unchallenged plants. **Figure S5.** Principal coordinate analysis (PCoA) with Bray-Curtis dissimilarity of the microbial community in bulk soil (B) or rhizosphere (R) of control (C) or pathogen-conditioned (P) soils using the whole ASV table excluding two ASVs belonging to the genera *Fictibacillus* and *Sphingomonas*, respectively. a) bulk soils; b) rhizosphere soils; c) all soil samples. **Table S1.** Soil properties in the control and pathogen-conditioned soil. **Table S2.** Relative abundance of two highly discriminative ASVs (*Fictibacillus* and *Sphingomonas*) in bulk soil samples from the control-conditioned (BC) versus pathogen-conditioned (BP) soils. **Table S3.** 200–300 ASVs together differentiate the microbial communities of pathogen conditioned and control bulks soil. (DOCX 690 kb)

Additional file 2: R code and root exudate compounds. (ZIP 360 kb)
