# Supplementary material for: Root exudates drive the soil-borne legacy of aboveground pathogen infection
Source: Microbiome. 2018 Sep 12;6:156. doi: 10.1186/s40168-018-0537-x (PMC6136170; doi:10.1186/s40168-018-0537-x)
Supplement: Supplementary file 1 — Figure S1. Schematic representation of the experimental design. Figure S2. Detection of Pseudomonas syringae pv. tomato strain DC3000 by PCR. The length of target fragment is 304 bp. Figure S3. Principal coordinate analysis (PCoA) with Bray-Curtis dissimilarity of the microbial community in bulk soil (B) or rhizosphere (R) of control (C) or pathogen-conditioned (P) soils. A) bulk soils, B) rhizosphere soils. Figure S4. A and B are the relative abundance (%) of the bacterial genera within the Firmicutes and Proteobacteria phyla in the microbial communities of control and pathogen- conditioned bulk soils, respectively. C: Relative abundance (%) of the major bacterial phyla using the whole ASV table excluding ten differential ASVs (Fictibacillus and Sphingomonas) present in the microbial communities of control (C) or pathogen-conditioned (P) soils. Samples were taken from the bulk (B) soil or rhizospheres (R) of unchallenged plants. Figure S5. Principal coordinate analysis (PCoA) with Bray-Curtis dissimilarity of the microbial community in bulk soil (B) or rhizosphere (R) of control (C) or pathogen-conditioned (P) soils using the whole ASV table excluding two ASVs belonging to the genera Fictibacillus and Sphingomonas, respectively. a) bulk soils; b) rhizosphere soils; c) all soil samples. Table S1. Soil properties in the control and pathogen-conditioned soil. Table S2. Relative abundance of two highly discriminative ASVs (Fictibacillus and Sphingomonas) in bulk soil samples from the control-conditioned (BC) versus pathogen-conditioned (BP) soils. Table S3. 200–300 ASVs together differentiate the microbial communities of pathogen conditioned and control bulks soil. (DOCX 690 kb) [file 40168_2018_537_MOESM1_ESM.docx]

**Additional file**

Figure S1

Schematic representation of the experimental design. Soils were conditioned by growing five consecutive generations of Arabidopsis plants in the same soil. The leaves of the plants in each of these generations were either inoculated with *Pseudomonas syringae* pv. *tomato* (*Pst*) or mock-treated. A sixth generation was then planted and used to assess effects of conditioning on either aboveground phytohormone levels and belowground microbiome composition or *Pst* disease resistance; In a second set of experiments (right side), root exudates of *in vitro* grown plants were analyzed by GC/MS. The effect of differentially released compounds on soil suppressiveness was subsequently investigated.


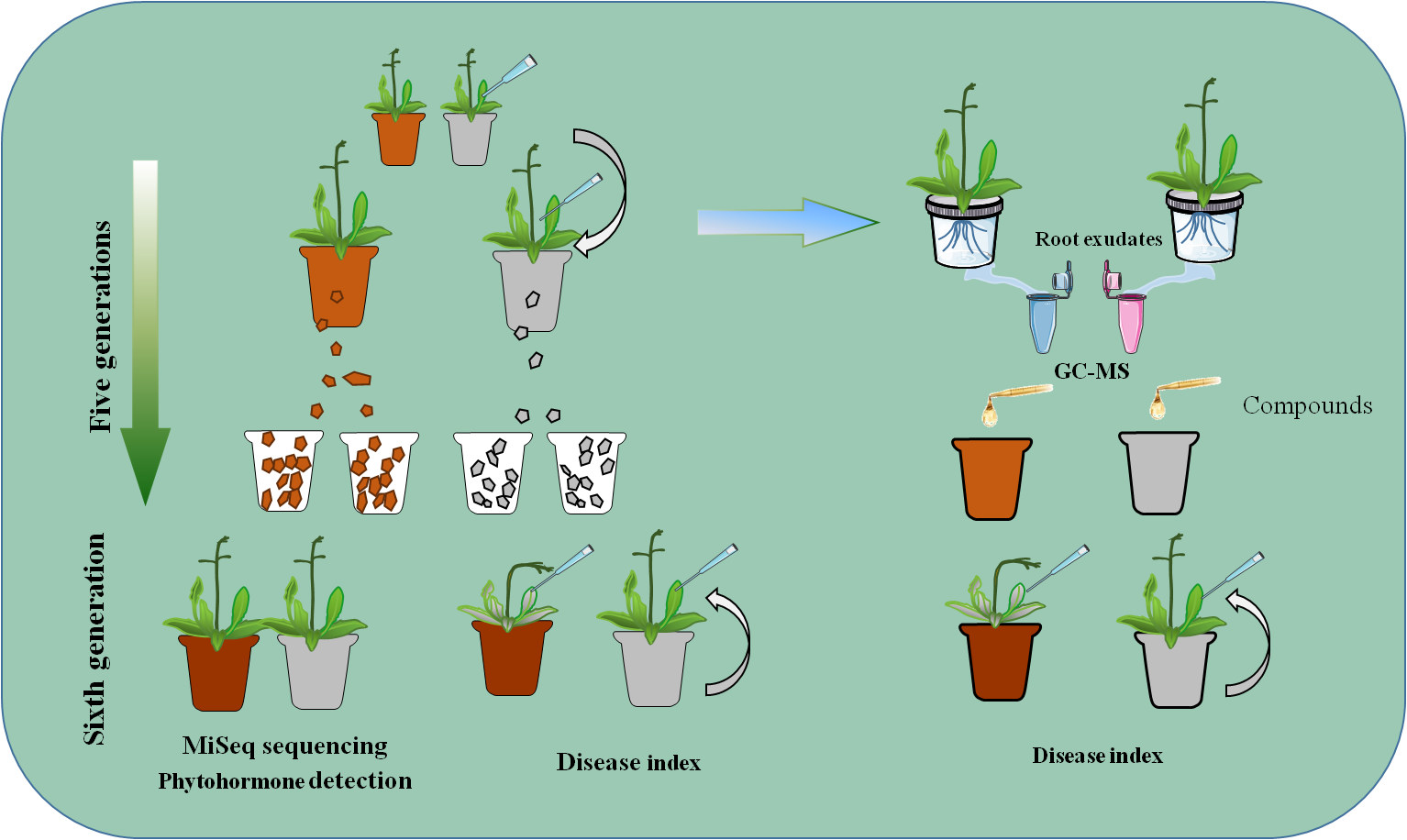


Figure S2

Detection of *Pseudomonas syringae* pv. tomato strain DC3000 by PCR. The length of target fragment is 304 bp.


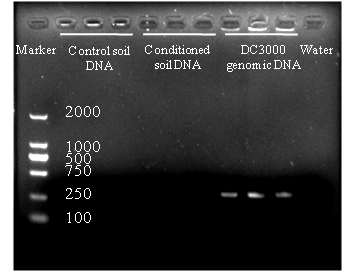


Figure S3

Principal coordinates analysis (PCoA) with Bray-Curtis dissimilarity of the microbial community in bulk soil (B) or rhizosphere (R) of control (C) or pathogen-conditioned (P) soils. **A)** bulk soils; **B)** rhizosphere soils.


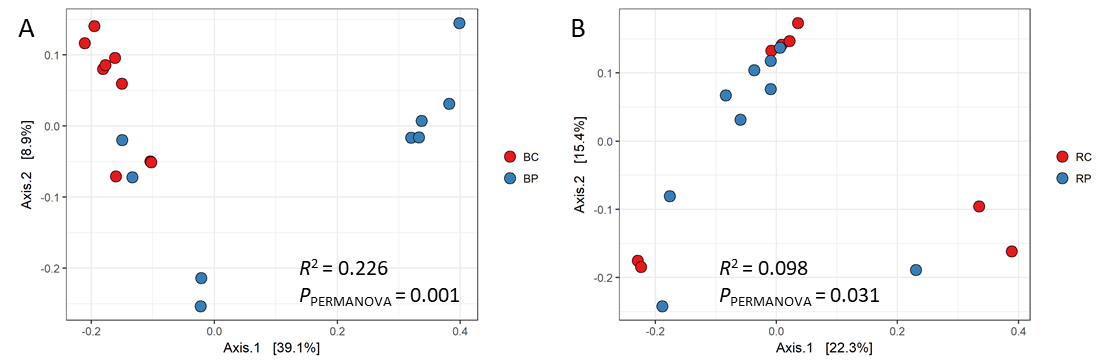


Figure S4

A and B are the relative abundance (%) of the bacterial genera within the *Firmicutes* and *Proteobacteria* phyla in the microbial communities of control and pathogen- conditioned bulk soils, respectively.

C: Relative abundance (%) of the major bacterial phyla using the whole ASV table excluding ten differential ASVs (*Fictibacillus* and *Sphingomonas*) present in the microbial communities of control (C) or pathogen-conditioned (P) soils. Samples were taken from the bulk (B) soil or rhizospheres (R) of unchallenged plants.


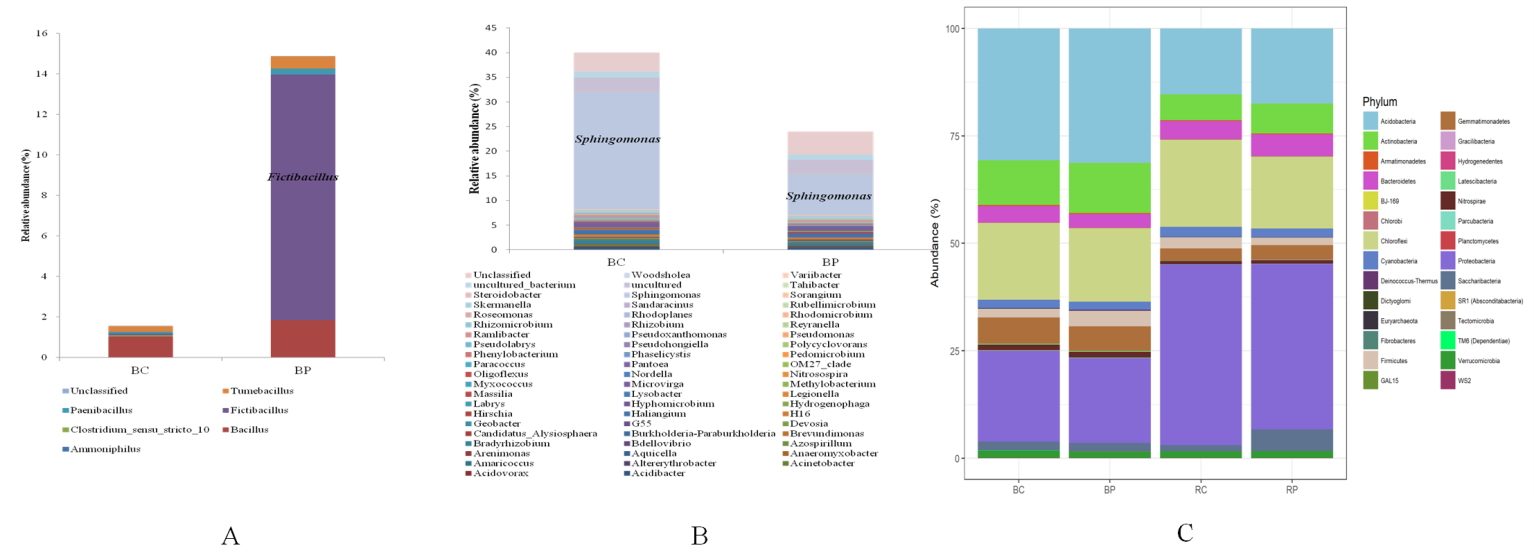


Figure S5

Principal coordinates analysis (PCoA) with Bray-Curtis dissimilarity of the microbial community in bulk soil (B) or rhizosphere (R) of control (C) or pathogen-conditioned (P) soils using the whole ASV table excluding two ASVs belonging to the genera *Fictibacillus* and *Sphingomonas*, respectively. **a)** bulk soils; **b)** rhizosphere soils; **c)** all soil samples


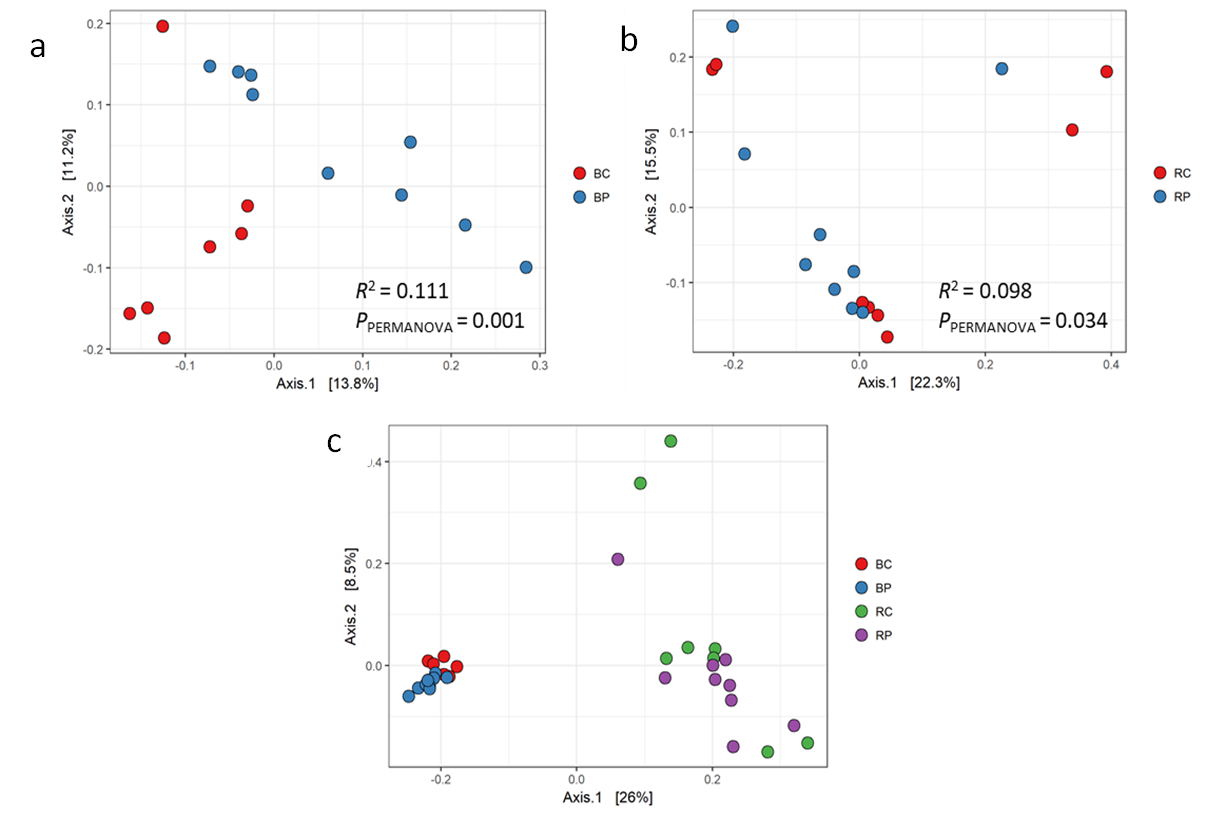


Table S1

Soil properties in the control and pathogen-conditioned soil.

| Treatment | pH | NO_3_^-^-N | | NH_4_^+^-N | available P | available K |
| --- | --- | --- | --- | --- | --- | --- |
|  |  |  | ppm | | | |
| Control | 7.2 ±0.1 | 3.3±0.7 | | 6.5±0.96 | 19.1±2 | 131.1±10.3 |
| Pathogen | 7.4 ±0.11 | 2.5±1.4 | | 6.9±1.56 | 19.7±0.6 | 125.1±6.8 |

T-test was used to examine the significance of differences and data show mean ± SD. No significant differences were observed between treatments.

Table S2

Relative abundance of two highly discriminative ASVs (*Fictibacillus* and *Sphingomonas*) in bulk soil samples from the control-conditioned (BC) versus pathogen-conditioned (BP) soils.

| Samples | Relative Abundance (%) | |
| --- | --- | --- |
|  | *Fictibacillus* | *Sphingomonas* |
| BC1 | 0 | 27.86 |
| BC2 | 0 | 28.44 |
| BC3 | 0.10 | 21.21 |
| BC4 | 0 | 27.44 |
| BC5 | 0 | 15.84 |
| BC6 | 0.16 | 19.12 |
| BC7 | 0 | 24.73 |
| BC8 | 0.17 | 22.46 |
| BC9 | 0.22 | 29.48 |
| BP1 | 25.44 | 1.68 |
| BP2 | 0 | 24.30 |
| BP3 | 0 | 21.63 |
| BP4 | 20.33 | 1.26 |
| BP5 | 17.57 | 1.41 |
| BP6 | 0 | 11.97 |
| BP7 | 23.40 | 1.53 |
| BP8 | 22.38 | 1.13 |
| BP9 | 0 | 11.56 |

Table S3. 200-300 ASVs together differentiate the microbial communities of pathogen conditioned and control bulks soil.

| # ASVs filtered from ASV table | *R*^2^ | *P* value |
| --- | --- | --- |
| 0 | 0.226 | 0.001 *** |
| 2 (*Fictibacillus* + *Sphingomonas*) | 0.111 | 0.001 *** |
| 50 | 0.087 | 0.001 *** |
| 100 | 0.083 | 0.006 ** |
| 200 | 0.077 | 0.021 * |
| 300 | 0.071 | 0.061 |
| 400 | 0.061 | 0.344 |

A large number of ASVs together contribute to the statistically significant difference in bacterial communities between control and pathogen-conditioned bulk soil-samples. Increasing numbers of ASVs were deleted from the bulk soil ASV table based on their ranked (from smallest to largest) *P* values in DESeq2 differential abundance analysis. These filtered ASV tables were then subjected to PERMANOVA based on their Bray-Curtis dissimilarity matrices. Although only 3 ASVs were significantly differentially abundant between the experimental groups in bulk soil samples, PERMANOVA produced a *P value* >0.05 only when the 300 most differentiating ASVs were removed from the analysis. Two ASVs (*Fictibacillus* and *Sphingomonas,* ranked 7 and 48 respectively in the DESeq2 differential abundance analysis) were found to drive the separation of five out of 9 pathogen-conditioned bulk soil samples in PCoA.
